# Supplementary material for: Rice OsMRG702 and Its Partner OsMRGBP Control Flowering Time through H4 Acetylation
Source: Int J Mol Sci. 2023 May 25;24(11):9219. doi: 10.3390/ijms24119219 (PMC10252469; doi:10.3390/ijms24119219)
Supplement: Supplementary file 1 [file ijms-24-09219-s001.zip › Supplemental figures.docx]

Supplementary Material


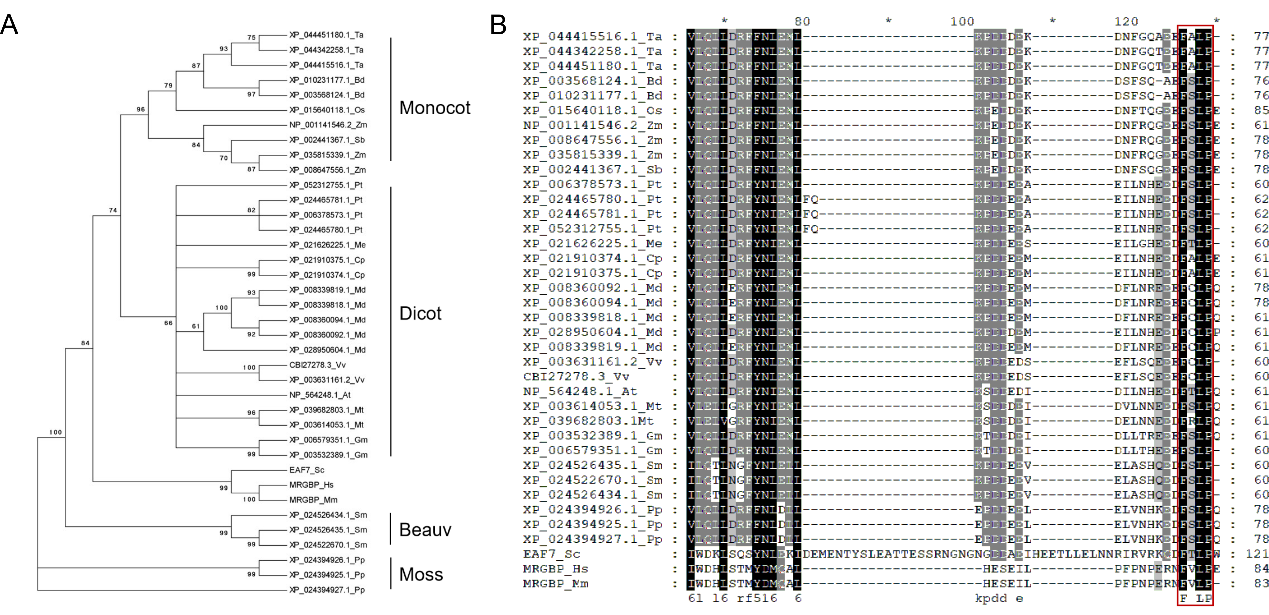


**Figure S1.** Phylogenic analysis of full-length of MRGBP homologous proteins (A) and alignment of the EAF7 domains (B) in plants and yeast (Sc), human (Hs) and mouse (Mm). Amino acid sequence numbers are marked by * and numbers. The conserved FxLP motif is highlighted by red box. Species abbreviations are as follows: At, *Arabidopsis* *thaliana*; Bd, *Brachypodium distachyon*; Cp, *Carica papaya*; Gm, *Glycine max*; Md, *Malus domestica*; Me, *Manihot esculenta*; Mt, *Medicago truncatula*; Os, *Oryza sativa L.*; Pp, *Physcomitrella patens*; Pt, *Populus trichocarpa*; Sb, *Sorghum bicolor*; Sm, *Selaginella moellendorffii*; Ta, *Triticum aestivum*; Vv, *Vitis vinifera* and Zm, *Zea mays*.

**
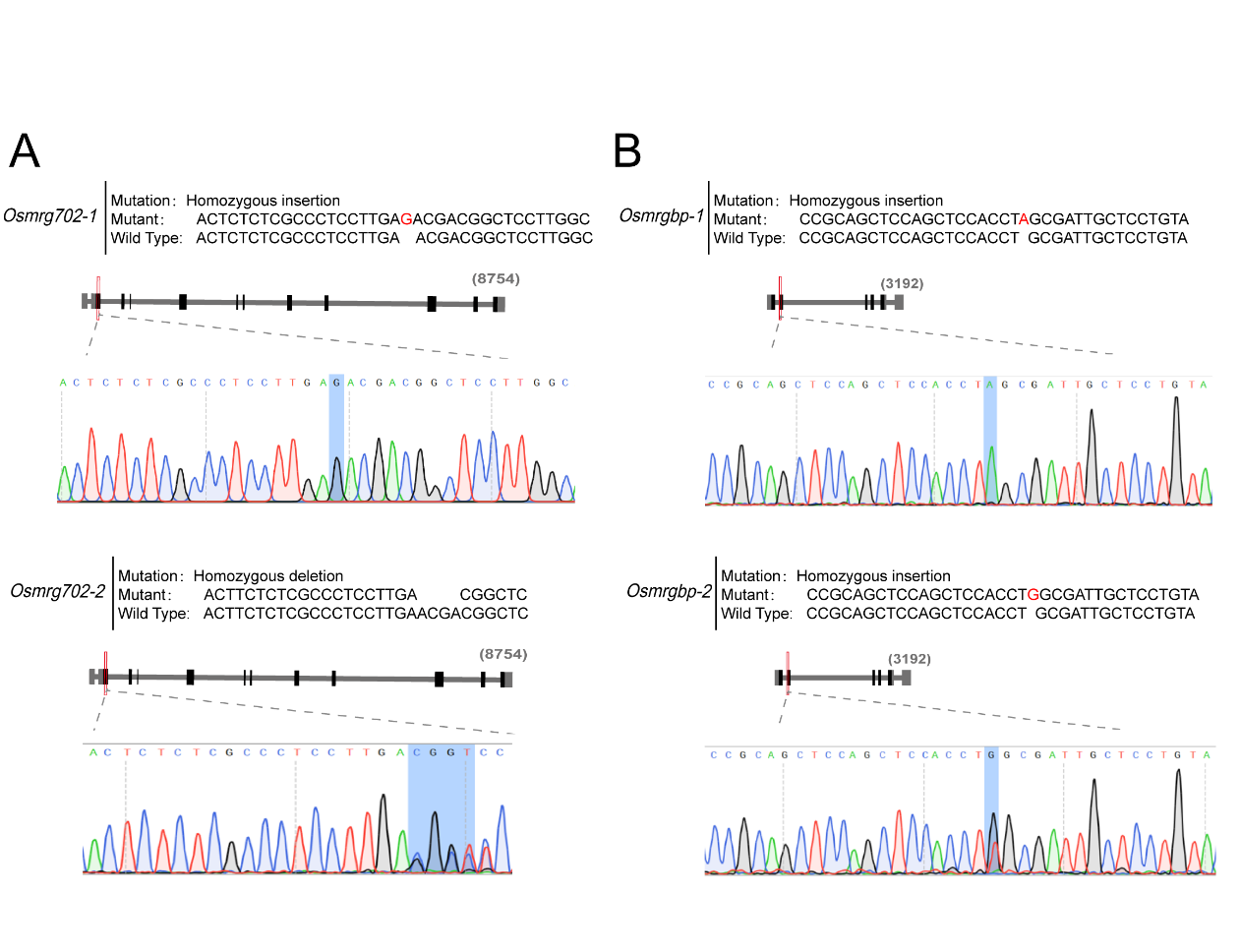
**

**Figure S2.** Mutated sequences at the target regions of *OsMRG702* (A) and *OsMRGBP* (B) in two individual lines. Red characters refer to base insertion, blank area in mutant means base depletion.


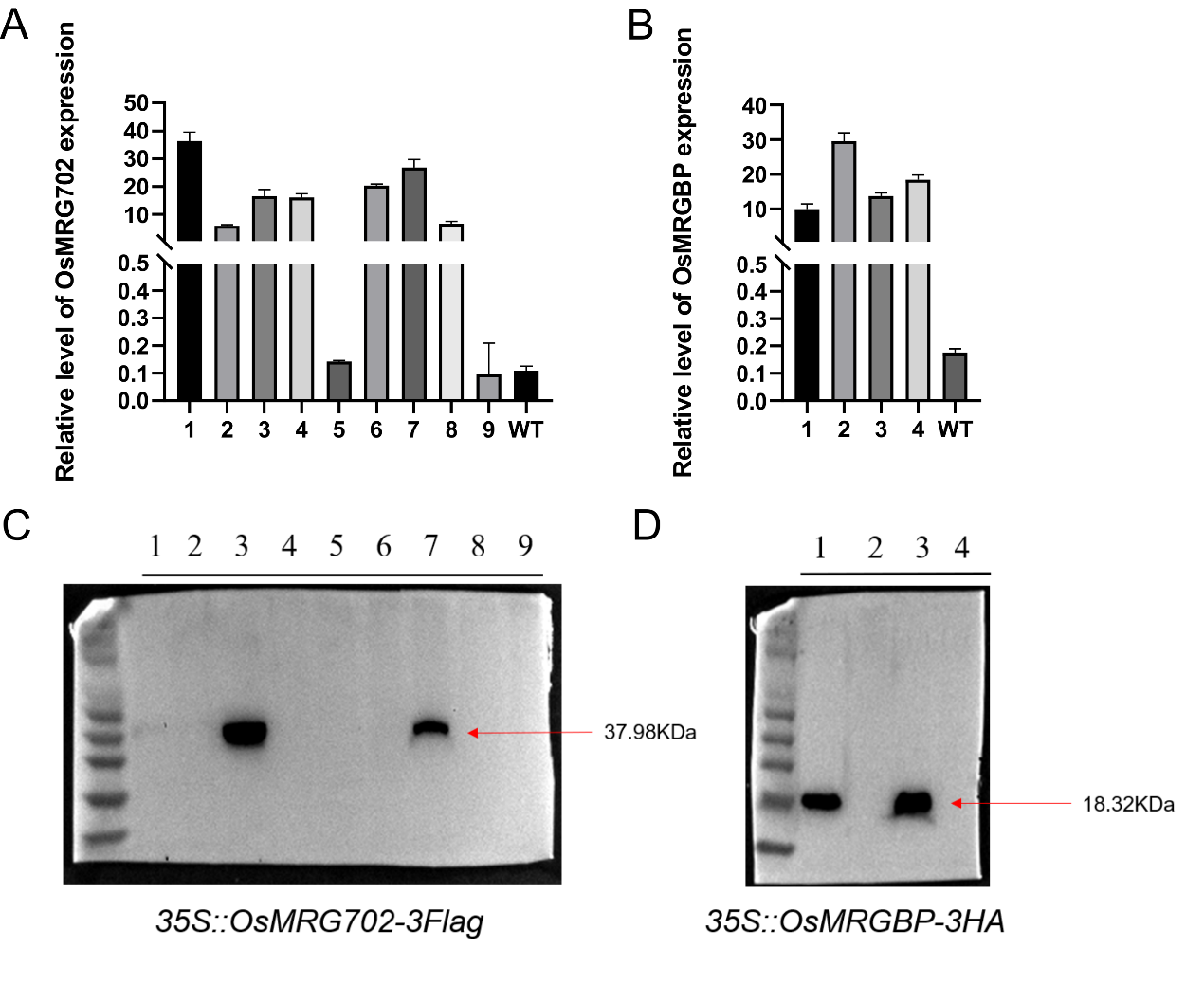


**Figure S3.** OsMRG702-3Flag and OsMRGBP-3HA overexpressing transgenic lines verification. (A-B) RT-qPCR to evaluate the transcripts of OsMRG702 and OsMRGBP in overexpressing lines. (C-D) Western blot analysis to verify the fusion protein in transgenic lines.

**
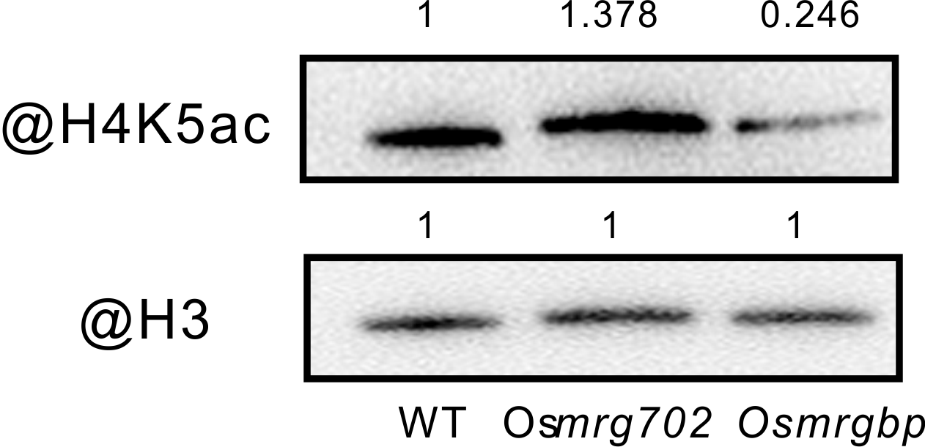
**

**Figure S4.** Global H4K5ac level is increased in *Osmrg702* but not in *Osmrgbp*. The signal intensity was quantified with the load nuclei protein amount normalized by H3.
